# Finite element analysis of cementless femoral stems based on mid- and long-term radiological evaluation

**DOI:** 10.1186/s12891-016-1260-z

**Published:** 2016-09-19

**Authors:** Kanehiro Matsuyama, Yasuhiro Ishidou, Yong-Ming Guo, Hironori Kakoi, Takao Setoguchi, Satoshi Nagano, Ichiro Kawamura, Shingo Maeda, Setsuro Komiya

**Affiliations:** 1 Department of Medical Joint Materials, Graduate School of Medical and Dental Science, Kagoshima University, 8-35-1 Sakuragaoka, Kagoshima, 890-8520 Japan; 2 Department of Mechanical Engineering, Graduate School of Science and Engineering, Kagoshima University, Kagoshima, Japan; 3 Department of Orthopaedic Surgery, Graduate School of Medical and Dental Science, Kagoshima University, Kagoshima, Japan; 4 The Near-Future Locomotor Organ Medicine Creation Course (Kusunoki Kai), Graduate School of Medical and Dental Science, Kagoshima University, Kagoshima, Japan

**Keywords:** Finite element analysis (FEA), Stress shielding, Cementless stem, Total hip arthroplasty (THA)

## Abstract

**Background:**

Femoral bone remodeling in response to stress shielding induces periprosthetic bone loss. Computerized finite element analysis (FEA) is employed to demonstrate differences in initial stress distribution. However, FEA is often performed without considering the precise sites at which the stem was fixed. We determined whether FEA reflects mid-term radiological examination exactly as predicted following long-term stress shielding.

**Methods:**

Femur–stem fixation sites were evaluated radiologically according to the location of spot welds in two anatomical cementless stem designs. Based on mid-term radiological results, four femur–stem bonding site conditions were defined as: (Condition A) no bonding; (Condition B) bonding within the 10 mm area proximal to the distal border of the porous area; (Condition C) bonding of the entire porous area; and (Condition D) bonding of the entire femoral stem, prior to conducting FEA analysis. Furthermore, we radiographically evaluated mid- and long-term stress shielding, and measured bone mineral density of the femur 10 years after total hip arthroplasty.

**Results:**

Spot welds appeared frequently around the border between the porous and smooth areas. FEA showed that, based on mid-term radiological evaluation, von Mises stress was reduced in condition B in the area proximal to the femur–stem bonding sites for both stem designs compared with condition A (no bonding). Conversely, von Mises stress at all areas of the femur–stem bonding sites in conditions C and D was higher than that in condition A. With respect to stress shielding progression, there was no significant difference between the two types of stem designs. However, stress shielding progressed and was significantly higher in the presence of spot welds (*p* = 0.001). In both stem designs, bone mineral density in zone VII was significantly lower than that in the contralateral hips.

**Conclusions:**

These results indicate that FEA based on mid-term radiological evaluation may be helpful to predict the influence of long-term stress shielding more precisely.

**Electronic supplementary material:**

The online version of this article (doi:10.1186/s12891-016-1260-z) contains supplementary material, which is available to authorized users.

## Background

Advances in stem surface processing technologies have led to improved fixation of cementless femoral stems following total hip arthroplasty (THA), resulting in better long-term outcomes [1–4]. A variety of cementless femoral stems are widely used, with excellent long-term outcomes [5]. Conversely, improvements in cementless implant fixation can result in stress shielding issues in the proximal femur. Femoral bone remodeling in response to stress shielding, incurred by altering the implant design, can induce periprosthetic bone loss [4, 6, 7]. Bone loss due to stress shielding is a key factor in aseptic loosening, subsidence and periprosthetic femoral fracture, making revision surgery difficult [8–10].

Computerized finite element analysis (FEA) is widely employed to demonstrate differences in initial stress distribution diversified by stem design, and to estimate stress shielding depending on periprosthetic bone remodeling [11–14]. However, FEA is often performed with the stem not bonded onto the femur, or with it completely bonded, without considering the sites at which the stem is fixed. In many studies, FEA was performed when the whole porous coated surface was bonded to the femur [15–18]. Broeke et al. reported that FEA in the fully bonded condition with proximal hydroxyapatite coating could not completely match the clinical findings [15]. Osseointegration between the implant and bone is not achieved immediately after THA and does not occur over the whole porous coated surface [16]. Scannell et al. describe that osseointegration occurs over a period of time and is determined by numerous factors [17]. Therefore, simulations should take this osseointegration process into account. These findings show that FEA prediction of long-term outcomes has not been achieved yet. Clinical data to help modulate the FEA conditions to make more accurate estimates of long-term clinical results are therefore needed.

Radiographic visuals, such as spot welds, predict fixation of cementless stems to bone by osseointegration [19]. FEA carried out without considering these in vivo bone reactive sites may not accurately predict the long-term outcome. Few reports have examined the influence of stem and femur bonding condition on FEA performance.

We radiologically evaluated femur–stem fixation sites according to the location of spot welds in two cementless stem designs. Based on mid-term radiological results, we defined four femur–stem bonding conditions and performed FEA. Furthermore, we radiographically evaluated mid- and long-term stress shielding, and measured bone mineral density (BMD) of the femur via dual energy X-ray absorptiometry (DEXA) 10 years after THA. We determined whether FEA exactly reflected mid-term radiological examination to predict long-term stress shielding.

## Methods

### Subjects

**Fig. 1:**
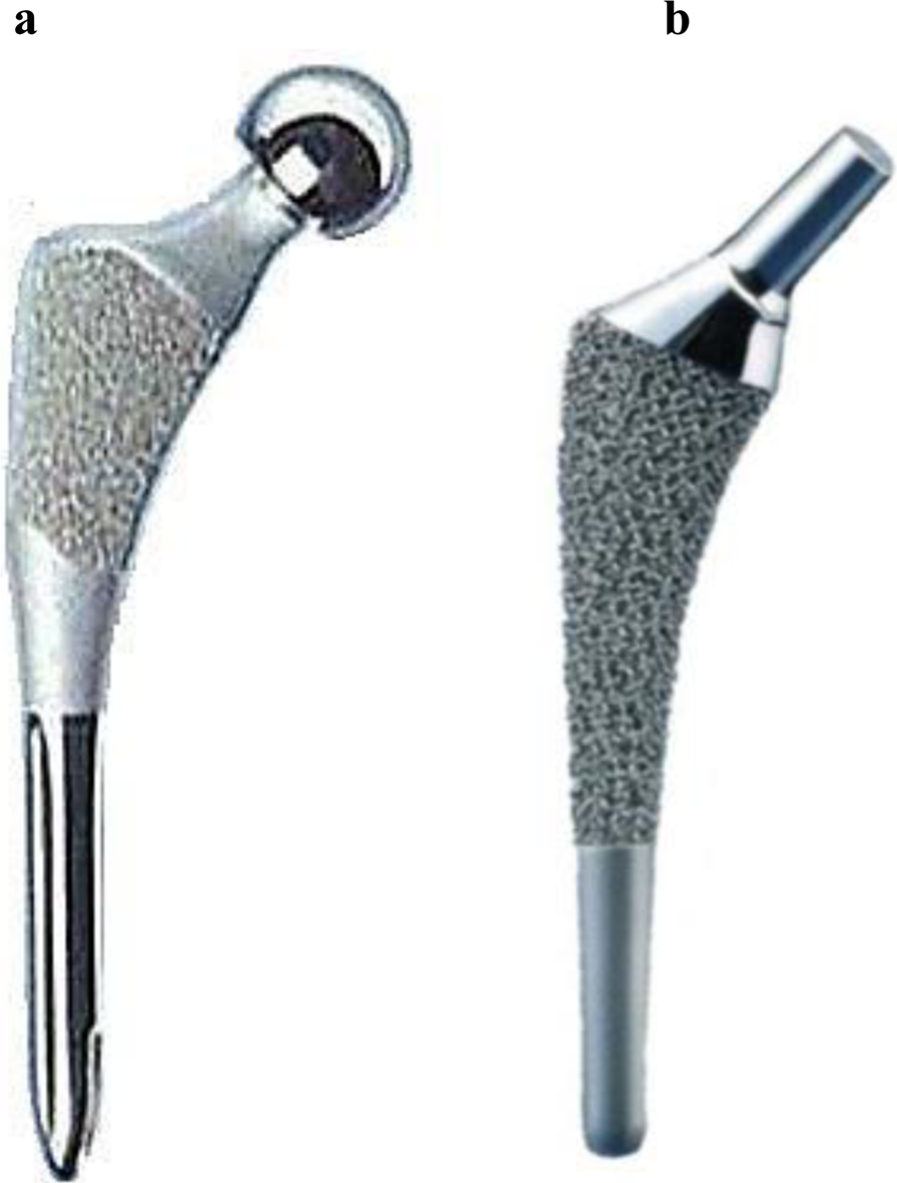
Cementless femoral stem designs. **a** Citation; **b** Spongiosa

We conducted a retrospective study in 41 patients (48 hips) who underwent THA and bipolar hemiarthroplasty with either Citation (Stryker, Tokyo, Japan) (Fig. 1a) or Spongiosa (Zimmer, Warsaw, IN, USA) (Fig. 1b) prostheses from 1999 to 2006. From these, a total of 31 patients (35 hips) were followed-up for more than 5 years postoperatively. The Citation prosthesis was used in 16 patients (16 hips: 4 male hips and 12 female hips; Group C) and the Spongiosa was used in 15 patients (19 hips: 7 male hips and 12 female hips; Group S). The mean age at surgery was 54.8 years (range, 34–76 years) in Group C and 58.4 years (range, 27–74 years) in Group S. In Group C, the preoperative diagnosis was osteoarthritis (OA) in 8 hips, osteonecrosis in 5 hips, rheumatoid arthritis in 2 hips, and femoral neck fracture in one hip. In Group S, the preoperative diagnosis was OA in 8 hips, osteonecrosis in 10 hips, and rheumatoid arthritis in one hip. There were no differences between Groups C and S with regard to sex, mean age at surgery, and preoperative diagnosis. Both prostheses were fit-and-fill type with an anatomic stem, and were made of CoCr alloy. However, porous structure (Citation: beads, Spongiosa: tripod), porous size (Citation: 425 μm, Spongiosa: 800–1500 μm) and porous area (Citation: proximal 33 % of the stem, Spongiosa: proximal 60 % of the stem) were different.

### Radiological evaluation of spot weld sites

Spot weld sites based on radiographs obtained 5–7 years after surgery were evaluated using Gruen’s classification of zones [20] by three orthopedic surgeons. Each zone, except zone IV, was divided into proximal and distal areas. Mean follow-up periods were 66.1 months (range, 60–74 months) and 66.3 months (range, 60–82 months) in Groups C and S, respectively. There was no difference between Groups C and S with regard to mean follow-up period.

### Finite element analysis

**Fig. 2:**
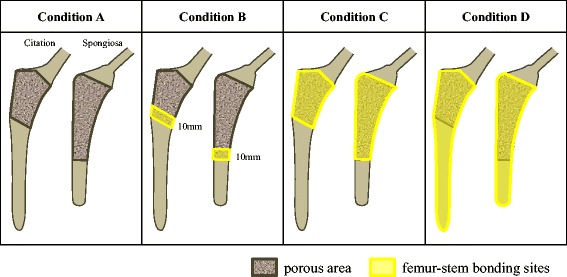
Four conditions of the femur–stem bonding sites in FEA. Condition A: no bonding between femur and stem; Condition B: bonding between femur and stem within the 10 mm area proximal to the distal border of the stem porous area; Condition C: bonding between the femur and entire porous area of the stem; Condition D: bonding between the femur and the entire area of the stem

The femur model was created based on a computed tomography (CT) scan of the right femur of a 60-year-old woman diagnosed with OA. Stem models of both prostheses were created based on stem samples (Citation: size 2; Spongiosa: size 2, both collarless) that were press-fit into the femur. For more precise expression of the complex three-dimensional forms, a mixture of 8-node hexahedral and 6-node pentahedral elements was used. Femur models were created to match both stem shapes. In the analysis of Citation, the femur model contained 1485 8-node hexahedral elements and 8 6-node pentahedral elements, and the stem model contained 7767 8-node hexahedral elements and 114 6-node pentahedral elements. In the analysis of Spongiosa, the femur model contained 1592 8-node hexahedral elements and 7 6-node pentahedral elements, and the stem model contained 6886 8-node hexahedral elements and 14 6-node pentahedral elements. Young’s modulus was set at 394 MPa for the cancellous bone [21], at 17.1 GPa for the femoral cortical bone [22], at 241 GPa for Citation, and at 210 GPa for Spongiosa. Poisson’s ratio was set at 0.12 for the cancellous bone and 0.3 for the cortical bone [23] for both prostheses. Regarding the friction coefficients of the interfascial bone surface and prosthesis, the friction coefficient of the porous area was set at 0.3 for Citation and 0.4 for Spongiosa, and the friction coefficient of the nonporous areas was set at 0.1 for both prostheses [24, 25]. Assuming the task of walking during daily living activities by a patient weighing 50 kg, the loading condition was set based on a study by Heller et al. [26, 27] at resultant forces of 1165 N (on the stem head), 512 N (abductor muscle force) and 464 N (vastus lateralis muscle force) to the grater trochanter. The distal end of the femur was fully fixed and based on radiographic evaluation the following four conditions for the femur–stem bonding sites were set (Fig. 2): (Condition A) no bonding between the femur and stem, assuming initial fixation of the stem; (Condition B) bonding between the femur and stem within the 10 mm area proximal to the distal border of the stem porous area, taking into account the site for the appearance of spot welds; (Condition C) bonding between the femur and entire porous area of the stem; (Condition D) bonding between the femur and the entire area of the stem. We used Marc Mentat 2013.0.0 software (MSC Software Japan, Tokyo, Japan) for the FEA.

### Radiological evaluation of stress shielding

We followed up 27 hips (11 in Group C; 16 in Group S) for approximately (~) 10 years postoperatively (range, 8 years 7 months–15 years 3 months). To assess the degree of stress shielding, Engh’s classification [28] was used for mid-term radiological evaluation ~5 years postoperatively (range, 5–6 years 3 months) and long-term evaluation ~10 years postoperatively. There were no differences between Groups C and S with regard to sex, mean age, diagnosis, and mean follow-up period at radiological evaluation of stress shielding.

### Evaluation of bone mineral density

BMD around the each stem and in the each contralateral femur was measured by DEXA (Hologic QDR Discovery W type, Toyo Medic., Tokyo, Japan) at ~10 years postoperatively. The region of interest was divided into seven Gruen zones. We evaluated the BMD measured by this method in 19 hips (9 in Group C, 10 in Group S) and 11 contralateral hips (6 in Group C, 5 in Group S). Operated hips, a dislocated hip, and a hip with bone atrophy due to paralysis were excluded in the contralateral hips. The mean age at BMD measurement was 66.8 years (range, 48–84 years) in the contralateral hips, 66.3 years (range, 48–80 years) in Group C and 67.1 years (range, 42–84 years) in Group S. There were no differences between Group C, Group S and the contralateral hips with regard to sex, mean age, diagnosis and mean follow-up period at BMD measurement.

### Statistical analysis

The Mann–Whitney *U* test was used for statistical analysis of the radiological evaluation of stress shielding between Groups C and S. The Tukey–Kramer test was used for statistical analysis of the evaluation of BMD between Group C, Group S and the contralateral hips. Mann–Whitney *U* test, one-way analysis of variance (ANOVA), and nonparametric Kruskal–Wallis test were used for statistical analysis of sex, mean age, preoperative diagnosis, and mean follow-up period between two groups (Group C and Group S) or three groups (Group C, Group S and the contralateral hips). The significance level was set at *p* < 0.05. Statistical analyses were performed using Statcel3 software (OMS, Saitama, Japan).

## Results

### Radiological evaluations of spot weld sites

**Fig. 3:**
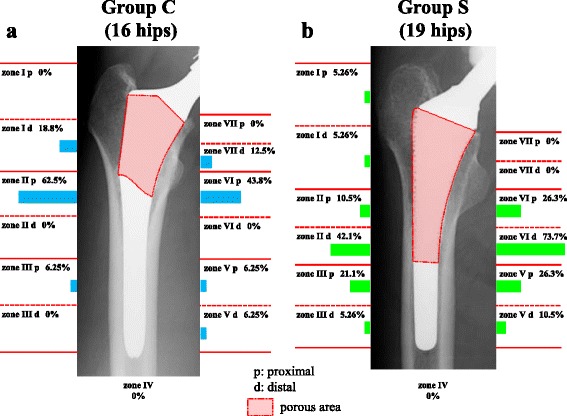
Locations and incidence of spot welds. **a** Group C; **b** Group S. Spot welds appeared frequently around the border between the porous and smooth areas in each group

Spot welds appeared in zone II proximal (62.5 %), zone VI proximal (43.8 %), zone I distal (18.8 %) and zone VII distal (12.5 %) in Group C (Fig. 3a, Table 1, Additional file 1), and in zone VI distal (73.7 %), zone II distal (42.1 %), zone V proximal (26.3 %), zone VI proximal (26.3 %), zone III proximal (21.1 %) and zone II proximal (10.5 %) in Group S (Fig. 3b, Table 1, Additional file 1). These data showed that spot welds appeared frequently around the border between the porous and smooth areas in each group. Furthermore, spot welds appeared around the border between the porous and smooth areas in 13 of 16 hips (81.3 %) in Group C and 15 of 19 hips (78.9 %) in Group S.

**Table 1:** Location and incidence of spot welds

Gruen’s zone		Group C (*n* = 16)	Group S (*n* = 19)
zone I	proximal	0.00 % (0)	5.26 % (1)
	distal	18.8 % (3)	5.26 % (1)
zone II	proximal	62.5 % (10)	10.5 % (2)
	distal	0.00 % (0)	42.1 % (8)
zone III	proximal	6.25 % (1)	21.1 % (4)
	distal	0.00 % (0)	5.26 % (1)
zone IV		0.00 % (0)	0.00 % (0)
zone V	proximal	6.25 % (1)	26.3 % (5)
	distal	6.25 % (1)	10.5 % (2)
zone VI	proximal	43.8 % (7)	26.3 % (5)
	distal	0.00 % (0)	73.7 % (14)
zone VII	proximal	0.00 % (0)	0.00 % (0)
	distal	12.5 % (2)	0.00 % (0)

### Finite element analysis

**Fig. 4:**
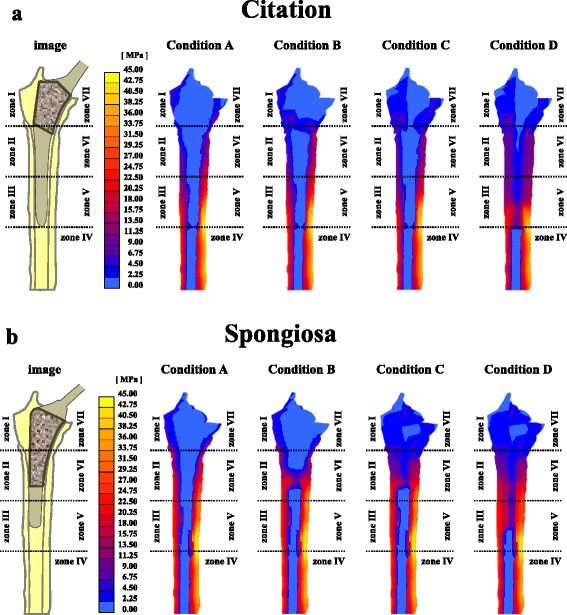
von Mises stress distribution at coronal section of the femur. **a** Citation; **b** Spongiosa

FEA was used to compare conditions B–D with condition A, assuming initial fixation of the stem in each condition. Analysis of the Citation (Fig. 4a) bonded areas found that von Mises stress in condition B was higher than that in condition A. In the area of zone VII, von Mises stress in condition B was lower than that in condition A. In the area of zone I proximal, von Mises stress in condition B was comparable to condition A. In the condition C bonded areas, (zone I and zone VII), von Mises stress was higher than that in condition A. In the condition D bonded areas (zone I and zone VII, as well as zone II–III and zone V–VI), von Mises stress was higher than that in condition A.

Analysis of the Spongiosa (Fig. 4b) bonded areas found that von Mises stress in condition B was higher than that in condition A. In the areas of zone II proximal, zone VI proximal, and zone VII, von Mises stress in condition B was lower than that in condition A. In the area of zone I, von Mises stress in condition B was comparable to condition A. In the condition C bonded areas (zone I–II and zone VI–VII), von Mises stress was higher than that in condition A. In the condition D bonded areas, (zone I-II and zone VI–VII, as well as zone III and zone V), von Mises stress was higher than that in condition A.

### Radiological evaluation of stress shielding

**Fig. 5:**
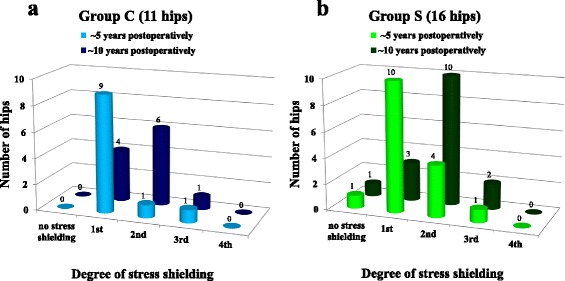
Comparison of stress shielding ~5 and ~10 years postoperatively. **a** Group C; **b** Group S. Stress shielding ~10 years postoperatively was intensified in ~50 % of hips in both groups compared with that ~5 years postoperatively

In Group C, the degree of stress shielding ~5 years postoperatively was first degree in 9 hips, second degree in 1 hip and third degree 1 hip. At ~10 years postoperatively, stress shielding was classified as first degree in 4 hips, second degree in 6 hips, and third degree in 1 hip (Fig. 5a, Table 2, Additional file 2). In Group S, the degree of stress shielding at ~5 years postoperatively was no stress shielding in 1 hip, first degree in 10 hips, second degree in 4 hips, and third degree in 1 hip. At ~10 years postoperatively, stress shielding was no stress shielding in 1 hip, first degree in 3 hips, second degree in 10 hips, and third degree in 2 hips (Fig. 5b, Table 3, Additional file 2).

**Table 2:** In Group C, comparison of stress shielding ~5 and ~10 years postoperatively

The degree of stress shielding	~5 years postoperatively	~10 years postoperatively
no stress shielding	0	0
1st	9	4
2nd	1	6
3rd	1	1
4th	0	0

**Table 3:** In Group S, comparison of stress shielding ~5 and ~10 years postoperatively

The degree of stress shielding	~5 years postoperatively	~10 years postoperatively
no stress shielding	1	1
1st	10	3
2nd	4	10
3rd	1	2
4th	0	0

Stress shielding progressed in 5 of 11 hips (45.5 %, 5 hips: from first to second degree) in Group C and in 8 of 16 hips (50.0 %, 7 hips: from first to second degree; one hip: from second to third degree) in Group S from 5 to 10 years postoperatively. At final observation, second-degree or greater stress shielding occurred in 7 of 11 hips (63.6 %) in Group C, and 12 of 16 hips (75.0 %) in Group S (Table 4, Additional file 3). There were no significant differences between Groups C and S with regard to the progression of stress shielding and the case of second-degree or greater stress shielding at final observation.

**Table 4:** Comparison of stress shielding between Groups C and S

	Group C	Group S	*P* value
Total number of hips	11	16	
Progression of stress shielding (ratio)^a^	5 (45.5 %)	8 (50 %)	0.820
Stress shielding ≥2nd degree (ratio)^b^	7 (63.6 %)	12 (75.0 %)	0.533

^a^Stress shielding progressed from ~5 years to ~10 years postoperatively

^b^Second-degree or greater stress shielding at ~10 years postoperatively

Because there were no significant differences between Groups C and S with regard to these findings, all 27 hips were assessed for the presence or absence of spot welds around the border between the porous and smooth areas and were compared for stress shielding (Table 5, Additional file 3). Stress shielding progressed in 12 of 21 hips (57.1 %) in which spot welds were present and in 1 of 6 hips (16.7 %) in which spot welds were absent. Stress shielding tended to progress in the presence rather than absence of spot welds, but the difference was not significant (*p* = 0.086). Second-degree or greater stress shielding was significantly greater in the presence (18 of 21 hips, 85.7 %) compared with absence (1 of 6 hips, 16.7 %) of spot welds at final observation (*p* < 0.01).

**Table 5:** Comparison of stress shielding between cases of the presence and absence of spot welds

	Presence of spot welds	Absence of spot welds	*P* value
Total number of hips	21	6	
Progression of stress shielding (ratio)^a^	12 (57.1 %)	1 (16.7 %)	0.086
Stress shielding ≥2nd degree (ratio)^b^	18 (85.7 %)	1 (16.7 %)	0.001

^a^Stress shielding progressed from ~5 years to ~10 years postoperatively

^b^Second-degree or greater stress shielding at ~10 years postoperatively

### Evaluation of bone mineral density

**Fig. 6:**
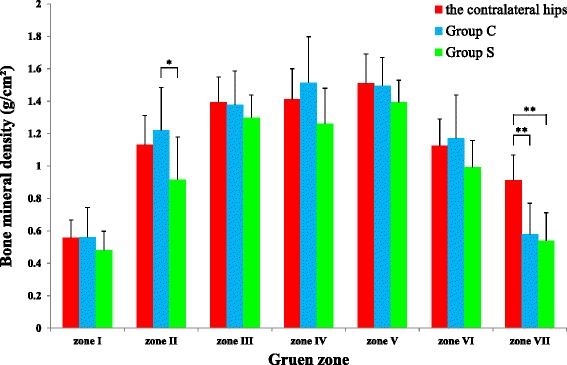
Comparison of mean BMD between Groups C and S and the contralateral hips. *p < 0.05; **p < 0.01

In zone VII, mean BMDs in Groups C and S were significantly lower than those in the contralateral hips (*p* < 0.01). Furthermore, in zone II, the mean BMD in Group S was significantly lower than that in Group C (*p* < 0.05). In all other zones, there were no significant differences in mean BMDs between Groups C and S and the contralateral hips (Fig. 6, Table 6, Additional file 4).

**Table 6:** Comparison of mean BMD between Groups C and S and the contralateral hips

		Gruen zone						
		I	II	III	IV	V	VI	VII
The contralateral hips	average (g/cm2)	0.557	1.131	1.393	1.412	1.509	1.126	0.913
	SD	0.110	0.180	0.155	0.188	0.183	0.165	0.155
Group C	average (g/cm2)	0.561	1.222	1.379	1.516	1.495	1.171	0.578
	SD	0.183	0.263	0.207	0.282	0.176	0.269	0.193
Group S	average (g/cm2)	0.481	0.917	1.298	1.261	1.393	0.994	0.541
	SD	0.117	0.262	0.141	0.219	0.137	0.164	0.171

## Discussion

The stability and durability of cementless femoral stems after THA are affected by periprosthetic bone remodeling. We radiologically determined the femur–stem bonding sites according to the location of spot welds in two designs of cementless stem and performed FEA in different bonding conditions. Furthermore, we examined the progression of stress shielding and BMD as a result.

Spot welds appeared most frequently around the border between the porous area and the smooth area in each group. We therefore postulated that the femur–stem bonding sites occurred within the area 10 mm proximal to the distal border of the stem’s porous area. FEA showed that von Mises stress under condition B, based on mid-term radiological evaluation, was reduced in the area proximal to the femur–stem bonding sites for both prostheses compared with that under condition A (no bonding). Conversely, von Mises stress in all areas of femur–stem bonding sites in conditions C and D was higher than that in condition A. These findings suggest that the stress shielding in condition B may be more intensified than that in conditions C or D.

We then investigated whether FEA under condition B, based on mid-term radiological evaluation, could determine the outcome 10 years after THA. With respect to the progression of stress shielding from 5 to 10 years after THA, there was no significant difference between the two stem designs. In addition, there was no significant difference in the degree of stress shielding between the two stem designs. Engh’s classification of stress shielding provides a rough evaluation, therefore the Group C versus Group S evaluation of stress shielding may not be sufficiently accurate [29]. However, stress shielding progressed and was significantly higher in the presence of spot welds around the border between the porous and smooth areas. These results were congruous to the FEA of condition B under which von Mises stress was reduced in the area proximal to femur–stem bonding sites.

In both stem designs, BMD in zone VII was significantly lower than that in the contralateral hip. FEA showed that stress shielding at zone VII was increased in conditions C and D compared with condition A, but was decreased compared with condition B. Therefore, condition B may be more appropriate than conditions C or D for predicting stress shielding. In addition, BMD in zone II was significantly lower in Group S than that in Group C. It is possible that stress shielding was more intensified in Group S than in Group C, particularly in zone II. These findings substantiated that the stem–femur fixation site in Group S was more distal than in Group C.

Regardless of bonding condition, von Mises stress was increased at the distal site of the cementless stems, and decreased proximally. Changes in stress distribution depend on stem stiffness, and stress shielding is known to influence current cementless stems [16, 30, 31]. Furthermore, the extent of stress shielding is affected by the bonding conditions of the implant–bone interface [32].

To reduce stress shielding, proximal femur–stem bonding is preferable to distal femur–stem bonding. Therefore, modification of the stem design has been attempted to reduce the porous coating area [33–35]. However, decreasing the extent of the porous coating alone does not necessarily reduce proximal femoral bone loss [36].

Femur–stem bonding was not identified in all porous areas. Spot welds, indicating bone ingrowth to the implant interface, were mainly located at the distal site of the porous area, not only in extensively-coated cases, but also in proximally-coated cases [36]. Interestingly, grid blasting onto the distal prosthesis part is known to result in bone remodeling at the boundary of the distal grid blasted and polished areas (zones 3 and 5), as well as at the proximal porous-coated area [37, 38].

Of the two stem designs assessed, the differences in their porous structures and coated surface areas did not significantly affect progression of stress shielding from 5 to 10 years. However, the presence or absence of spot welds did affect progression of long-term stress shielding. These findings indicate that stress shielding is affected by the bonding conditions of the implant–bone interface.

Measurement of BMD can evaluate the impact of stress shielding [39]. In the present study, the only significant difference between the two stem designs, owing to the different porous areas, was in zone II 10 years postoperatively. Because porous structure was different from Citation in Spongiosa, we set the different friction coefficient for each stems in FEA. The change of the von Mises stress by the difference in the friction coefficient might modify the influence of the different range of porous area, therefore it was difficult to predict the difference of BMD results in the two stem designs by FEA. Setting the same friction coefficient in FEA or comparing in the same porous designs may be needed to predict the differences of BMD influenced by range of porous area using FEA.

Our results suggested that not only was the initial stem-cortical bone contact area affected by stem design, but also the later stem–femur bonding area due to bone ingrowth, both of which should be taken into account when predicting the long-term outcome of cementless stems.

Spot welds were not always observed following THA, and their locations varied between individual cases. Preoperative BMD is one major factor that influences periprosthetic bone loss [40]. Post-operative bone loss was also observed in the late phase 5 years or more after surgery [37].

Preoperative factors that influence postoperative bone remodeling around the stem include prosthetic-related (materials [41–44], shape [45, 46], size [47–50], extent of porous area [35, 41, 47, 48]) and patient-related (BMD [40, 49, 51, 52], sex [53, 54], bone canal shape [55–57]). These preoperative factors may not be sufficient to predict long-term radiological outcomes. It is therefore essential to evaluate bone remodeling around the stem several years after operation and to use these findings to determine FEA conditions. Taken together, our results indicate that FEA based on mid-term radiological evaluation, may be helpful to predict the influence of long-term stress shielding more precisely.

Although the present study verified FEA could be used to evaluate changes in the stem–femur bonding conditions, it could not be used to directly compare Citation and Spongiosa. Consequently, the effects of porous area should be verified using the same stem design. Several limitations exist in this study. First, BMD was not measured over time, therefore we could not exactly define bone loss from preoperative bone quality. Second, we used a representative femoral bone model obtained from one CT database case. Consequently, the defined stem–femur bonding sites based on spot welds are not necessarily consistent with the radiological findings of all patients. To predict personal outcomes in individual cases, FEA modified by individual personal data would be needed.

## Conclusions

Our results suggest that not only was the initial stem-cortical bone contact area affected by stem design but also by the later stem–femur bonding area due to bone ingrowth, both of which should be taken into account to predict long-term outcomes of cementless stems. FEA based on mid-term radiological evaluation may be helpful to predict the influence of long-term stress shielding more precisely.

## Additional files

Additional file 1: Locations and incidence of spot welds (Fig. 3). (XLSX 10 kb) Additional file 2: Comparison of stress shielding ~5 and ~10 years postoperatively (Fig. 5). (XLSX 12 kb) Additional file 3: Comparison of stress shielding (Tables 4 and 5). (XLSX 10 kb) Additional file 4: Comparison of mean BMD between Groups C and S and the contralateral hips (Fig. 6). (XLSX 13 kb)

## Abbreviations

BMD: Bone mineral density
CT: Computed tomography
DEXA: Dual energy X-ray absorptiometry
FEA: Finite element analysis
OA: Osteoarthritis
THA: Total hip arthroplasty
